# Clinicopathological Spectrum of Breast Cancer Patients Presenting at a Tertiary Care Hospital in Sindh, Pakistan

**DOI:** 10.7759/cureus.86249

**Published:** 2025-06-17

**Authors:** Sara Khalid Memon, Faiza Hameed, Nawaz A Dal, Mahak W Qureshi, Qambar A Laghari

**Affiliations:** 1 Department of Surgery, Liaquat University of Medical and Health Sciences, Jamshoro, PAK

**Keywords:** breast cancer, breast cancer imaging, hormone receptors, primary breast malignancy, south asian population

## Abstract

Objective

The objective of this study was to evaluate the various clinicopathological characteristics of breast cancer patients presenting at the Department of Surgery at Liaquat University Hospital, Jamshoro.

Study design and setting

A cross-sectional analytical study was conducted at the Department of Surgery, Liaquat University Hospital, Jamshoro, Pakistan, from 1 November 2024 to 30 April 2025.

Methodology

With a population size of around 150 patients, a margin of error of 10%, and a CI of 99%, 79 patients were asked to fill out a structured questionnaire using a consecutive sampling technique. Data were analyzed using SPSS version 16 (SPSS Inc., Chicago, IL, USA). The frequency of various clinicopathological characteristics was measured using descriptive analytics.

Results

A total of 79 patients were included, with a mean age of 41.94±10.85. Most women were married for more than 20 years and have breastfed their children for more than 2 years. Also, most of the patients had early menarche. The average size of the tumor was 2.08 cm, with the most common occurrence being in the upper outer quadrant (64.46%). Out of 79 patients, 36 (45.6%) had nipple retraction and 71 (89.9%) had palpable axillary lymph nodes. Metastatic disease was seen in 7 out of 79 (8.9%) patients.

Among pathological parameters, 72 (91.1%) patients had intraductal carcinoma, and 50 out of 79 (63.3%) patients had a pathological grade III tumor. The majority of patients, 28 (35.4%), had estrogen receptor (ER)/progesterone receptor (PR)-positive and human epidermal growth factor receptor 2 (HER2/neu)-negative disease, whereas the second common presentation is of triple negative (ER, PR, HER2/neu) tumors, observed in 15 (19%) patients.

Conclusion

This study highlights the clinical and pathological parameters of breast cancer disease in patient presenting in this region of Pakistan. This will help us in developing the guidelines regarding risk factors, clinical signs, and hormonal clues present in breast cancer patients in the area of Pakistan for early diagnosis and management of breast cancer disease.

## Introduction

Breast cancer is the main cause of death among women, following lung cancer [1]. Though the incidence has been decreasing recently in developed countries, it is still growing rapidly and is an unstoppable issue in third-world countries like Pakistan. There have been multiple associations with the pathogenesis of this disease, i.e., family history, estrogen exposure, exposure to radiation, previous history of abnormal hyperplasia, western-style diet, including more saturated fats, alcohol consumption, and more [2]. According to diagnostic protocols, evaluation of a suspected breast mass involves a triple assessment approach: clinical examination (including palpation of the breast and axilla), imaging (with ultrasound and mammography), and histopathological confirmation through a core needle biopsy [3]. About a portion of breast tumors occur in ladies who have no recognizable breast disease risk factor other than gender (female) and age more than 40 years [4]. Despite the fact that symptomatic standards has progressed in different bearings, one-third of a hundred thousand females pass on malignant growth of breast consistently [5], which is dependent on a number of variables, such as late diagnosis, the type of tumor, the number of lymph nodes involved at the time of tumor diagnosis, the size and grade of the tumor, the presence of the cellular marker for proliferation Ki67, the patient's age, the presence of the p53 mutation, and the presence of the human epidermal growth factor receptor 2 (HER2/neu) overexpression [6]. One more essential job in this manner is being played by the breast cancer awareness programs, which are still missing in reaching the underdeveloped areas. Recent studies have focused on assessing hormonal status and gene overexpression in breast cancer patients, particularly those with triple-positive or triple-negative profiles. For instance, a study conducted a few years ago in Karachi, Pakistan, reported that 76 out of 118 primary breast cancer patients were HER2/neu positive. Among these, 72.6% were premenopausal and 55.4% were postmenopausal, with clinical features found to be associated with HER2/neu expression [7]. HER2/neu overexpression has been associated with many malignancies such as breast, ovarian, lung, gastric, and oral cancers and particularly in one-third of breast cancers [8]. This association with breast cancer is not only established in cases reported from the Western world but also in third-world countries like Pakistan, although supporting literature is much less [9]. The purpose of this study is to observe the various clinical and pathological parameters in breast cancer patients admitted at the surgical ward of Liaquat University Hospital, Jamshoro.

## Materials and methods

After taking ethical approval from the institutional review board, this study was conducted in the Department of Surgical Unit 01, Liaquat University Hospital, Jamshoro. It was a cross-sectional analytical study conducted for six months, from November 2024 to April 2025. The sample size was calculated using the OpenEpi software. With a population size of around 150 patients (anticipated frequency of 55.4% according to stats taken from the parent study) [6], a margin of error of 10%, and a CI of 99%, the sample size came out to be 79, calculated by the consecutive sampling technique. Patients with biopsy-proven breast cancer, with availability of their hormonal receptors, i.e., estrogen receptor (ER), progesterone receptor (PR), and HER2/neu, and in an age group between 15 and 60 years were included in the study. Patients with recurrent breast cancer, inflammatory breast disease, and phyllodes tumors, or those with primary breast cancer whose hormonal status was not determined were excluded from the study. All patients with breast cancer and a borderline HER2/neu score were also excluded from the study. Primary breast cancer patients with significant comorbidities, and those with contributing risk factors such as smoking, uncontrolled diabetes, and hypertension were also excluded from the study.

Data were collected using a standard proforma, which included all the important variables following the triple assessment scheme, i.e., detailed history, examination, radiological, and histopathological evidence. Data were entered and analyzed using SPSS version 16 (SPSS Inc., Chicago, IL, USA). Frequency and percentages were calculated. The mean and standard deviation were calculated for the ordinal variables of the patient.

## Results

A total of 79 patients with a mean age of 41.94±10.85 were enrolled in this study. Most women were married for more than 20 years and have breastfed their children for more than 2 years. Also, most of the patients had early menarche. The average size of the tumor was 2.08 cm, with the most common occurrence in the upper outer quadrant (64.46%). Out of 79 patients, 36 (45.6%) had nipple retraction, and 71 (89.9%) had palpable axillary lymph nodes. Metastatic disease was seen in 7 out of 79 (8.9%) patients (Tables 1, 2).

**Table 1:** Descriptive statistics showing categorical and ordinal variables based on information obtained from patients through inquiry

Ordinal variables	Mean	Standard deviation	95% confidence interval for the mean	
			Lower bound	Upper bound
Age (years)	41.94	10.85	39.51	44.37
Size of tumor (cm)	2.08	0.79	1.90	2.25

Categorical variables	Frequency	Percentage
Married since (years) (n=72)		
≤10	22	30.6%
11-20	21	29.2%
>20	29	40.3%
Menarche status		
Early	72	91.1%
Late	7	8.9%
Menopausal status		
Early	14	17.7%
Late	15	19.0%
Non-menopausal	50	63.3%
Lactational duration		
Complete 2 years	57	72.2%
Less than 2 years (incomplete)	22	27.8%

**Table 2:** Descriptive statistics showing clinical signs of breast cancer patients

Variables	Frequency	Percentage
Nipple retraction	36	45.6%
Peau d'orange/other skin signs	7	8.9%
Palpable axillary lymph nodes	71	89.9%
Metastasis	7	8.9%
Lump site		
Upper outer	51	64.6%
Lower outer	21	26.6%
Upper inner	7	8.9%
Surface of lump		
Regular	22	27.8%
Irregular	57	72.2%
Fixity of lump		
Mobile	36	45.6%
Fixed to chest wall	22	27.8%
Fixed to skin	21	26.6%

Among pathological parameters, 72 (91.1%) patients had intraductal carcinoma, and 50 out of 79 (63.3%) had pathological grade III tumor. The majority of patients, 28 (35.4%), had ER/PR-positive and HER2/neu-negative disease, whereas the second common presentation was of triple negative (ER, PR, HER2/neu) tumors, observed in 15 (19%) patients (Table 3).

**Table 3:** Descriptive statistics showing different histopathological parameters of the patients

Variables	Frequency	Percentage
Histopathological type of tumor		
Intraductal	72	91.1%
Intralobular	7	8.9%
Tumor size		
≤2 cm	22	27.8%
2.1 to 5 cm	29	36.7%
>5 cm	28	35.4%
Stages of the tumor		
Stage I	22	27.8%
Stage II	21	26.6%
Stage III	15	19.0%
Stage IV	21	26.6%
Grade of tumor		
Grade I	14	17.7%
Grade II	15	19%
Grade III	50	63.3%
Hormonal stage of biopsy		
ER +	7	8.90%
ER and PR +	28	35.4%
ER, PR, and HER2/neu +	22	27.8%
HER2/neu +	7	8.90%
ER, PR, and HER2/neu -	15	19.0%

## Discussion

Breast cancer is among the most commonly diagnosed malignancies and remains a leading cause of cancer-related mortality in women worldwide. It accounts for approximately 23% of new cancer cases and 14% of cancer-related deaths globally [10]. In accordance with figures, developing countries account for almost half of breast cancer diagnoses and 60% of the death toll [11]. Pakistan likewise bears a gigantic burden of cancer-related disease and has the highest incidence of breast cancer in Asia. In Pakistan, multicenter studies have revealed that breast cancer is the most well-known harmful cancer and accounts for approximately 25% of all threatening growths in the female population [12]. Notably, proto-oncogenes and tumor suppressor genes play a vital part in the regulation of cell development and differentiation. Subsequently, any alteration in one or more of these genes appears to play a significant role in the pathogenesis of most human malignancies [13]. One of the most significant changes observed in breast cancer pathogenesis is HER-2/neu proto-oncogene amplification and/or overexpression. Gene expression analysis studies have determined the luminal A, luminal B, HER2/neu-enriched, and triple-negative subtypes of breast cancer as the four intrinsic molecular subtypes [14,15].

Recently, many researchers have focused on genes associated with the incidence and progression of breast cancer. In this study, 79 patients diagnosed with primary breast cancer confirmed on histopathology and with hormonal status (ER, PR, HER2/neu) were included. The frequency of breast cancer increases significantly with age, peaking around menopause, and then gradually declines or remains steady [16]. Breast cancer tends to be more aggressive in older populations due to late diagnosis and compromised immunity. Studies have attempted to correlate HER2/neu overexpression with clinical features of the disease. Our study showed a mean age of 41.94±10.85 years (range 15-60). A previous case-control study found an association between breast cancer incidence and age over 50 years [17], whereas another study in Saudi Arabia observed a similar trend beginning after 40 years [18]. Clinical variables such as age, parity, lactation, and pathological markers (tumor type, grade, and hormonal receptors) were assessed. Recent literature reports HER2/neu overexpression in 9%-40% of breast cancers, most commonly around 20% [16,19-21]. Emi et al. found no significant difference in age, tumor size, TNM staging, or lymph node involvement between HER2-positive and HER2-negative patients [22]. Furthermore, recent studies have shown a significant association between HER2/neu-positive breast cancers and higher recurrence rates [23,24].

## Conclusions

This study shows various signs, symptoms, and pathological parameters present in breast cancer patients in this part of the Pakistani population, which serves as a stepping stone in establishing risk factors, clinical factors, and prognostic factors for the South Asian population, in particular, in the management of breast cancer disease.

## References

[1] Ahmad A (2019). Breast cancer statistics: recent trends. Adv Exp Med Biol.

[2] McTiernan A (2003). Behavioral risk factors in breast cancer: can risk be modified?. Oncologist.

[3] Senkus E, Kyriakides S, Ohno S (2015). Primary breast cancer: ESMO clinical practice guidelines for diagnosis, treatment and follow-up. Ann Oncol.

[4] (2025). Breast cancer. https://www.who.int/news-room/fact-sheets/detail/breast-cancer.

[5] Greenlee RT, Murray T, Bolden S, Wingo PA (2000). Cancer statistics, 2000. CA Cancer J Clin.

[6] Soares MC, Rodrigues IJM, Almeida ICT da S de, Assunção JVP, Monteiro AM, Dias Júnior LB (2020). Histopathological and immunohistochemical parameters of breast cancer cases analyzed in a reference laboratory. Mastology.

[7] Shaikh F, Jamal Q, Baig S, Hadi NI, Majeedet N (2016). Correlation of hormone receptor and HER2/neu expression with clinicopathologic parameters in primary breast tumors. Asian Pac J Cancer Prev.

[8] (2025). Correlation between hepcidin and procalcitonin and their diagnostic role in patients with COVID-19. https://www.researchgate.net/profile/Alyaa-Alsaedi-2/publication/370254936_CORRELATION_BETWEEN_HEPCIDIN_AND_PROCALCITONIN_AND_THEIR_DIAGNOSTIC_ROLE_IN_PATIENTS_WITH_COVID-19/links/6448459a017bc07902db1a65/CORRELATION-BETWEEN-HEPCIDIN-AND-PROCALCITONIN-AND-THEIR-DIAGNOSTIC-ROLE-IN-PATIENTS-WITH-COVID-19.pdf.

[9] Ahmad A, Bano U, Gondal M, Khan A (2009). Her-2/neu gene overexpression in breast carcinoma and its association with clinicopathological characteristics of the disease. J Coll Physicians Surg Pak.

[10] Zhang L, Zhou X, Sha H, Xie L, Liu B (2022). Recent progress on therapeutic vaccines for breast cancer. Front Oncol.

[11] Asif HM, Sultana S, Akhtar N, Rehman JU, Rehman RU (2014). Prevalence, risk factors and disease knowledge of breast cancer in Pakistan. Asian Pac J Cancer Prev.

[12] Kondov B, Milenkovikj Z, Kondov G (2018). Presentation of the molecular subtypes of breast cancer detected by immunohistochemistry in surgically treated patients. Open Access Maced J Med Sci.

[13] Hashmi AA, Mahboob R, Khan SM (2018). Clinical and prognostic profile of Her2neu positive (non-luminal) intrinsic breast cancer subtype: comparison with Her2neu positive luminal breast cancers. BMC Res Notes.

[14] Gulzar R, Shahid R, Saleem O (2018). Molecular subtypes of breast cancer by immunohistochemical profiling. Int J Pathol.

[15] Al-Thoubaity FK (2020). Molecular classification of breast cancer: a retrospective cohort study. Ann Med Surg (Lond).

[16] Wilkinson L, Gathani T (2022). Understanding breast cancer as a global health concern. Br J Radiol.

[17] Nascimento RG do, Otoni KM (2020). Histological and molecular classification of breast cancer: what do we know?. Mastology.

[18] Arafah M (2012). Correlation of hormone receptors with Her-2 neu protein expression and the histological grade in invasive breast cancers in a cohort of Saudi Arabia. Turk Patoloji Derg.

[19] Mwakigonja AR, Lushina NE, Mwanga A (2017). Characterization of hormonal receptors and human epidermal growth factor receptor-2 in tissues of women with breast cancer at Muhimbili National Hospital, Dar es Salaam, Tanzania. Infect Agent Cancer.

[20] Fabi A, Di Benedetto A, Metro G (2011). HER2 protein and gene variation between primary and metastatic breast cancer: significance and impact on patient care. Clin Cancer Res.

[21] Rydén L, Landberg G, Stål O, Nordenskjöld B, Fernö M, Bendahl PO (2008). HER2 status in hormone receptor positive premenopausal primary breast cancer adds prognostic, but not tamoxifen treatment predictive, information. Breast Cancer Res Treat.

[22] Emi Y, Kitamura K, Shikada Y, Kakeji Y, Takahashi I, Tsutsui S (2002). Metastatic breast cancer with HER2/neu-positive cells tends to have a morbid prognosis. Surgery.

[23] Hashmi AA, Bukhari U, Najam J (2023). Luminal B, human epidermal growth factor receptor 2 (HER2/neu), and triple-negative breast cancers associated with a better chemotherapy response than luminal A breast cancers in postneoadjuvant settings. Cureus.

[24] Neuman HB, Schumacher JR, Edge SB (2023). The influence of anatomic stage and receptor status on first recurrence for breast cancer within 5 years (AFT-01). Cancer.

